# Specific regulation of muscle protein metabolism in broilers by dietary fermented cottonseed meal

**DOI:** 10.3389/fvets.2026.1774608

**Published:** 2026-03-05

**Authors:** Lianqing Wei, Yun Xu, Ruoyu Zhang, Boda Wang, Shuaijiang Guo, Yuxia Wang, Minghong Huang, Li Zhang, Wenxia Ge

**Affiliations:** 1 Xinjiang Agricultural Vocational Technical University, Changji, Xinjiang, China; 2 Key Laboratory of Biological Resources and Ecology of the Pamir Plateau, College of Life and Geographical Sciences, Kashgar University, Kashgar, Xinjiang, China

**Keywords:** fermented cottonseed meal, IGF-1/mTOR, muscle deposition, protein metabolism, white-feather broilers

## Abstract

This study aimed to investigate the regulatory effects of replacing soybean meal with varying proportions of fermented cottonseed meal in the diet on protein metabolism in the pectoral and leg muscles of white-feather broilers. The experiment was divided into two phases: early-growth (1–21 days) and late-growth (22–42 days) phases. The control group was fed a basal diet without fermented cottonseed meal (0%), whereas the experimental groups were fed soybean meal replaced with 3, 6, and 9% fermented cottonseed meal. The effect on protein metabolism was evaluated by measuring the expression of key genes in the IGF1/mTOR, AMPK, and UPP pathways in muscle tissue. During days 1–21, experimental groups showed significantly higher IGF-1 and mTOR mRNA expression in leg muscles compared to controls, while FoxO3 expression in pectoral muscles was reduced. The 3 and 6% groups had elevated IGF-1 and PI3K, and the 6% group showed higher AKT and mTOR in pectoral muscles and PI3K in leg muscles. TSC2 expression was suppressed in the 3 and 6% groups, while Atrogin-1 and MuRF1 were lowest in the 6% group. During days 22–42, the 3 and 9% groups had increased IGF-1 and mTOR in pectoral muscles, whereas the 6% group showed higher AKT and PI3K in leg muscles. FoxO3 was significantly downregulated in the 9% group. In summary, fermented cottonseed meal can regulate protein metabolism in the pectoral and leg muscles of Cobb broilers by enhancing the expression of genes related to the IGF-1/mTOR pathway and suppressing the transcription of certain key genes in the UPP, thereby promoting protein deposition, with an optimal supplementation level of 6%.

## 1 Introduction

Chicken is the second most consumed meat product in China after pork. Chicken quality primarily depends on the types, content, and composition of proteins, fats, and other trace components (1). In recent years, nutritional interventions have become a key research focus for improving meat quality, attracting widespread academic attention. However, studies on nutritional approaches to regulate muscle protein metabolism, particularly the role of fermented feed, remain limited. Cottonseed meal, as a high-quality protein feed ingredient, has restricted application efficiency owing to the presence of toxic substances such as free gossypol and multiple anti-nutritional factors (2). Microbial fermentation treatment can effectively enhance the nutritional value of cottonseed meal and degrade anti-nutritional factors (3, 4), thereby improving livestock utilization efficiency. Feeding fermented cottonseed meal helps to reduce abdominal fat accumulation (5), and increases total amino acids, inosine, and flavor amino acids (such as aspartic acid, glutamic acid, and glycine) in muscles, thereby improving meat quality and flavor (6). Previous studies have demonstrated that replacing soybean meal with fermented cottonseed meal can improve broiler growth and slaughter performance (7) and it promotes the metabolism of carcass fat and protein, with its effects being associated with the dosage of the additive (8, 9). Skeletal muscle, as the body’s largest protein reserve organ, maintains a dynamic equilibrium between synthesis and catabolism. Although fermented cottonseed meal can regulate broiler’s overall protein metabolism, the gene expression-related mechanism through which it controls this process remains unclear. Therefore, in this study, we investigated the effects of fermented cottonseed meal on protein metabolism in pectoral and leg muscles of broiler chickens from the perspective of protein synthesis and decomposition, and the expression of key genes in the PI3K-AKT–mTOR and adenosine monophosphate-activated protein kinase (AMPK) signaling pathways. The study holds significant practical value for improving feed-utilization efficiency, reducing breeding costs, and promoting the production of green and healthy livestock and poultry products.

## 2 Materials and methods

### 2.1 Preparation of fermented cottonseed meal

The cottonseed meal, corn, and bran used in this experiment were purchased from Xinjiang Taikun Feed Technology Co., Ltd. The fermentation strain employed was *Candida tropicalis* ZD-3 (ZD-3), a tropical yeast strain developed by the Feed Science Research Institute of Zhejiang University. The fermentation substrate consisted of cottonseed meal, corn, and bran in a 90:5:5 ratio, with a mass-to-water ratio of 1:0.8. After thorough mixing, the mixture underwent autoclaving. Once cooled, the sterilized substrate was inoculated under sterile conditions with an 8% fermentation broth (concentration: 2.97 × 10^9^ CFU/mL) and incubated aerobically at 30 °C for 48 h. Thereafter, the samples were dried in a 45 °C oven, ground, and filtered through a 60-mesh sieve for storage. The preparation of the microbial broth and substrate fermentation procedures followed the method described by Nie et al. (5).

### 2.2 Experimental design

This study used 480 one-day-old male Cobb broilers that were randomly divided into four treatment groups, with six replicates per group and 20 chickens per replicate. The control group received a basal diet without fermented cottonseed meal (designated as the 0% group), whereas each experimental group received soybean meal substituted with 3, 6, and 9% fermented cottonseed meal, respectively. All diets were formulated as powdered compound feeds according to the NRC (1994) nutritional requirements (10), ensuring consistent energy and crude protein levels between the control and experimental groups. Specific dietary compositions and nutritional levels are detailed in a previous study (8), with variations in key nutrients and amino acid profiles after fermented cottonseed meal substitution, as described previously (11). The chickens were housed in individual cages per replicate, provided with ad libitum access to feed and water, and followed standard immunization schedules. The experiment was divided into two phases: early growth (1–21 days) and late growth (22–42 days). The dietary compositions and nutritional levels for each phase are presented in Table 1.

**Table 1:** Composition and nutritional levels in experiment basal diets (based on air-dried feed).

Items	1–21 days of age				22–42 days of age			
	0%	3%	6%	9%	0%	3%	6%	9%
Corn	54.50	54.50	54.30	54.30	58.20	58.20	58.15	58.00
Soybean meal	33.50	30.40	27.40	24.30	28.80	25.70	22.65	19.60
Fermented cottonseed meal	0.00	3.00	6.00	9.00	0.00	3.00	6.00	9.00
Sunflower oil	3.00	3.10	3.30	3.40	4.00	4.10	4.20	4.40
Cottonseed protein	4.00	4.00	4.00	4.00	4.00	4.00	4.00	4.00
Premix^1^	5.00	5.00	5.00	5.00	5.00	5.00	5.00	5.00
Nutrient content^2^								
Metabolizable energy (Mcal/kg)	2.95	2.95	2.95	2.95	3.06	3.05	3.05	3.05
Crude protein	21.23	21.21	21.23	21.21	19.52	19.51	19.52	19.51
Calcium	1.04	1.03	1.03	1.03	0.96	0.96	0.96	0.95
Phosphorus	0.69	0.70	0.71	0.72	0.64	0.65	0.66	0.67
Available phosphorus	0.45	0.45	0.45	0.46	0.41	0.41	0.41	0.42
Methionine	0.50	0.50	0.50	0.50	0.48	0.48	0.48	0.48
Methionine + Cysteine	0.86	0.86	0.86	0.86	0.82	0.82	0.82	0.82
Threonine	0.78	0.77	0.76	0.74	0.71	0.70	0.69	0.67
Lysine	1.12	1.09	1.08	1.05	1.00	0.98	0.96	0.94

^1^Premix provided the following per kg of diets: NaCl, 2.94 g/kg; Lys, 0.234 g/kg; Met, 1.791 g/kg; VA, 8800 IU/kg; VD, 3000 IU/kg; VE, 30 mg/kg; VK_2_, 1.65 mg/kg; VB_1_, 2.5 mg/kg; VB_2_, 6.6 mg/kg; VB_3_, 11 mg/kg; VB_4_, 500 mg/kg; VB_5_, 60 mg/kg; VB_6_, 4.0 mg/kg; VH, 0.2 mg/kg; VB_11_, 1.0 mg/kg; VB_12_, 0.02 mg/kg; VC, 50 mg/kg; Fe^2+^, 80.0 mg/kg; Cu^2+^, 8.0 mg/kg; Zn^2+^, 60.0 mg/kg; Mn^2+^, 70.0 mg/kg; I^−^, 0.5 mg/kg; and Se^4+^, 0.3 mg/kg. 1–21 d; Ca, 8.106 g/kg; P, 2.98 g/kg and 22–42 d Ca, 7.415 g/kg; P, 2.64 g/kg. ^2^All the nutritional components are measured values except for the metabolizable energy, which is a calculated value.

### 2.3 Parameter measurement and sample collection

#### 2.3.1 Production performance evaluation

During the trial period, detailed records of feed intake were maintained for each group. Fasting body weights were recorded at the start and end of each phase (specifically on days 1, 21, and 42). Average daily feed intake (ADFI), average daily gain (ADG), and feed-to-gain ratio (F/G) were calculated on a per-replicate basis.

#### 2.3.2 Measurement of slaughter performance

On the mornings of days 21 and 42, after fasting for 12 h, two chickens were randomly selected from each replicate. The chickens were slaughtered by neck vein bleeding, then immersed in hot water (approximately 75 °C) for scalding and feather removal. After drying the carcasses, the weights of the whole carcass, half of the carcass, pectoral muscle, and leg muscles were measured. Slaughter performance indicators, including slaughter rate, whole-carcass rate, half-carcass rate, pectoral muscle rate, and leg muscle rate, were calculated.

#### 2.3.3 Sample collection

On days 21 and 42 of the experiment, one chicken was randomly selected from each replicate (n = six per group) for sampling in the morning. After venous bloodletting, the muscle samples from the pectoral and legs were immediately collected, placed in cryopreservation tubes, and rapidly frozen in liquid nitrogen before being transported to the laboratory for storage at −80 °C.

### 2.4 Experimental methods

#### 2.4.1 Total RNA extraction from tissues

The pectoral and leg muscle samples stored at −80 °C in liquid nitrogen were homogenized. Total RNA was extracted using RNA extraction kit (Quan Shi Jin Biotechnology Co., Ltd.) according to the manufacturer’s instructions. The extracted RNA was purified and quantified using Nanodrop 2000 spectrophotometer (Thermo Fisher Scientific). Samples were deemed suitable for subsequent experiments only when the optical density (OD) ratio of OD_260_/OD_280_ was within the range of 1.8–2.0.

#### 2.4.2 Complimentary DNA (cDNA) synthesis and real-time quantitative PCR

cDNA was synthesized using the EasyScript^®^ One-Step gDNA Removal and cDNA Synthesis SuperMix Kit (QuanShiJin Biotechnology Co., Ltd.), according to the manufacturer’s protocol, and resulting products were stored at −20 °C. The mRNA expression levels of insulin-like growth factor (IGF-1), phosphatidylinositol-3-hydroxylase (PI3K), protein kinase B (AKT), mammalian target of rapamycin (mTOR), AMPK, atrophin-1 (Atrogin-1), myosin ring finger 1 (MuRF1), forkhead transcription factor (FoxO1/FoxO3), and tumor suppressor factor (TSC1/TSC2) were detected using the ABI 7500 real-time PCR system with *β*-actin as internal reference, and the TransStart^®^ Tip Green qPCR SuperMix Kit (QuanShiJin Biotechnology Co., Ltd.), following the manufacturer’s instructions. The reaction mixture (20 μL) was composed as follows: 2 × TransStart^®^ Tip Green qPCR SuperMix 10.0 μL, 0.5 μL each of forward and reverse primers, 1.0 μL cDNA template, and 8.0 μL ddH₂O. The reaction procedure was as follows: 95 °C pre-denaturation for 30 s; followed by 40 cycles of amplification (95 °C denaturation for 5 s, 50–60 °C annealing for 30 s, 72 °C extension for 30 s). The primer sequences used are listed in Table 2.

**Table 2:** Primer information of genes used in RT-qPCR.

Genes name	Primer sequence (5′-3′)	Annealing temperature (°C)	Product length/base pairs
*IGF-1*	Upstream: GTGTGGTGCTGAGCTGGTTGATG	60	110
	Downstream: CCTTGTGGTGTAAGCGTCTACTGC		
*mTOR*	Upstream: AACCACTGCTCGCCACAATGC	58	120
	Downstream: CATAGGATCGCCACAGGATTAGC		
*PI3K*	Upstream: TGCTCCGTAGTGGTAGACGG	58	185
	Downstream: CGCTTCATCGCCTCTGTTGT		
*AKT*	Upstream: GCCTGCCCTTCTAC AACCA	52	198
	Downstream: ACGATGCTGGCGAAGAAA		
*MuRF1*	Upstream: ATCTGGCTTGATTCCGGACG	56	179
	Downstream: TGGAAGATGTCGTTGGCACA		
*Atrogin-1*	Upstream: CCATAAGGAGAAGTGGATCTATGT	57	104
	Downstream: GCTTCCCCCAAAGTGCAGTA		
*FOXO1*	Upstream: TACGGCCAATCCAGCAT	55	154
	Downstream: TGGGGAGGAGAGTCAGAAGT		
*FOXO3*	Upstream: CGGCTCACTTTGTCCCAGAT	57	165
	Downstream: TCTTGCCAGTCCCTTCGTTC		
*AMPK*	Upstream: CACAGGCATATGGTGGTCCACAG	58	143
	Downstream: TCCACACAGCAAAGCATAGAG		
*TSC1*	Upstream: GAGTGACCGCGGATTAGAGG	59	199
	Downstream: GAAGGGAGAGTCAAAGCCCC		
*TSC2*	Upstream: CCTGTCCTTCCAACCCTCAC	59	189
	Downstream: GATACGGCTCTCTTCACCCG		
*β-actin*	Upstream: ATTGTCCACCGCAAATGCTTC	56	193
	Downstream: AATAAAGCCATGCCAATCTCGT		

All the primers above were designed by ourselves.

### 2.5 Statistical analysis

The experimental data were preliminarily processed in Excel, and the relative expression levels of the target gene were calculated using the 2^-ΔΔCt^ method (12). One-way analysis of variance (ANOVA) was performed using SPSS software (version 17.0), and least significant difference method (LSD) was employed for inter-group comparisons. Graphs were generated using GraphPad Prism 5. A significance level of *p* < 0.05 was set.

## 3 Results

### 3.1 Variations in nutritional parameters of cottonseed meal pre- and post-fermentation

As presented in Table 3, the fermented cottonseed meal group exhibited a significantly higher crude protein content than the control group (*p* < 0.05). In addition, crude fat and free gossypol concentrations were significantly lower in the fermented cottonseed meal group compared with all other experimental groups (*p* < 0.05). Moreover, viable colonies of *Candida tropicalis* were detected in the fermented cottonseed meal group at a concentration of 2.13 × 10^6^ CFU/g.

**Table 3:** Variations in nutritional parameters of cottonseed meal pre- and post-fermentation (DM basis) %.

Items	Cottonseed meal	The same treatment but without *Candida tropicalis*	Fermented cottonseed meal	*p*-value
Crude protein	38.39 ± 1.18^b^	38.41 ± 0.87^b^	44.20 ± 0.86^a^	<0.001
Dry matter	91.47 ± 0.26	91.41 ± 0.23	91.75 ± 0.27	0.083
Ether extracts	4.20 ± 0.34^a^	4.02 ± 0.19^a^	3.23 ± 0.44^b^	<0.001
Crude ash	6.03 ± 0.33	6.04 ± 0.44	6.58 ± 0.0.56	0.086
Free gossypol /(mg/kg)	151.28 ± 0.98^a^	149.64 ± 1.29^a^	29.50 ± 1.93^b^	<0.001
Calcium	0.28 ± 0.01	0.29 ± 0.03	0.31 ± 0.04	0.363
Phosphorus	0.69 ± 0.02	0.71 ± 0.03	0.74 ± 0.05	0.095
*Candida tropicalis*/(CFU/g)	/	/	2.13 × 10^6^ ± 2.05	<0.001

The results are expressed as mean and standard error. In the same row, values with different small letter superscripts mean significant different (*p* < 0.05), while with the same or no letter superscripts mean no significant different (*p* < 0.05).

### 3.2 Effects of fermented cottonseed meal on growth performance of white-feather broilers

As shown in Table 4, the supplementation of fermented cottonseed meal significantly affected the feed/gain ratio (F/G) during days 1–21, with the 6% supplementation group exhibiting a significantly lower F/G than the other groups (*p* < 0.05). The 9% group was significantly higher than the 3 and 6% groups. However, there was no significant difference compared with the control group (*p* < 0.05). From days 22–42, The average daily feed intake of 6% was significantly higher than that of the control group (*p* < 0.05). No significant differences were observed in other production parameters among all groups.

**Table 4:** The effect of fermented cottonseed meal on the growth performance of white-feather broilers.

Items	Control	Group 3%	Group 6%	Group 9%	*P*-value
1–21 days					
ADFI (g)	60.04 ± 5.72	61.34 ± 5.11	61.35 ± 4.32	59.87 ± 5.10	0.928
ADG (g)	41.48 ± 4.69	43.15 ± 4.24	45.22 ± 4.47	40.21 ± 4.25	0.259
F/G^1^	1.45 ± 0.03^ab^	1.42 ± 0.03^b^	1.36 ± 0.04^c^	1.49 ± 0.04^a^	<0.001
22–42 days					
ADFI (g)	150.78 ± 1.17^b^	152.08 ± 0.88^b^	156.10 ± 1.55^a^	147.99 ± 1.27^c^	<0.001
ADG (g)	88.52 ± 1.02	89.02 ± 5.14	89.17 ± 2.41	88.83 ± 3.58	0.989
F/G	1.71 ± 0.03	1.71 ± 0.10	1.75 ± 0.07	1.67 ± 0.07	0.273

^1^The abbreviation “F/G” denotes the ratio between the amount of feed consumed and the resulting body weight gain. The results are expressed as mean and standard error. In the same row, values with different small letter superscripts mean significant different (*p* < 0.05), while with the same or no letter superscripts mean no significant different (*p* < 0.05).

### 3.3 Effects of fermented cottonseed meal on the slaughter performance of white-feather broilers

As shown in Table 5, during days 1–21, the supplementation of fermented cottonseed meal significantly enhanced the slaughter rate, total eviscerated weight, pectoral muscle percentage, and leg muscle percentage compared with the control group (*p* < 0.05). Notably, the 3 and 6% supplementation groups exhibited significantly higher slaughter rates and total eviscerated weights than both the control group and the 9% supplementation group, with the 6% group demonstrating superior pectoral and leg muscle percentages (*p* < 0.05). During days 22–42, the 6% supplementation group showed a significantly higher slaughter rate and pectoral muscle percentage than the control group and other experimental groups (*p* < 0.05), whereas no statistically significant differences were observed for the remaining parameters.

**Table 5:** The effect of fermented cottonseed meal on the slaughter performance of white-feather broilers.

Items	Control	Group 3%	Group 6%	Group 9%	*P*-value
21 days					
Dressing percentage	85.70 ± 0.24^b^	86.10 ± 0.23^a^	86.30 ± 0.26^a^	85.54 ± 0.22^b^	<0.001
Percentage of eviscerated yield	70.86 ± 0.72^b^	73.00 ± 0.76^a^	72.50 ± 0.74^a^	71.50 ± 0.71^b^	<0.001
Percentage of pectoral muscle	20.84 ± 0.28^b^	21.49 ± 0.41^a^	22.06 ± 0.72^a^	21.65 ± 0.61^a^	0.007
Percentage of leg muscle	16.88 ± 1.32^b^	18.69 ± 1.55^a^	19.79 ± 1.37^a^	18.45 ± 1.39^ab^	0.019
42 days					
Dressing percentage	90.04 ± 0.87^b^	90.30 ± 1.05^b^	91.51 ± 1.09^a^	90.02 ± 0.83^b^	0.047
Percentage of eviscerated yield	80.93 ± 0.97	81.03 ± 0.75	81.26 ± 0.95	80.41 ± 0.49	0.349
Percentage of pectoral muscle	25.81 ± 0.92^b^	26.70 ± 0.57^ab^	27.08 ± 0.56^a^	25.82 ± 0.90^b^	0.017
Percentage of leg muscle	20.07 ± 2.17	21.01 ± 1.09	21.20 ± 0.24	20.45 ± 1.49	0.511

The results are expressed as mean and standard error. In the same row, values with different small letter superscripts mean significant different (*p* < 0.05), while with the same or no letter superscripts mean no significant different (*p* < 0.05).

### 3.4 Effects of fermented cottonseed meal on IGF-1/mTOR pathway in protein synthesis metabolism of white broiler chickens

**Figure 1:**
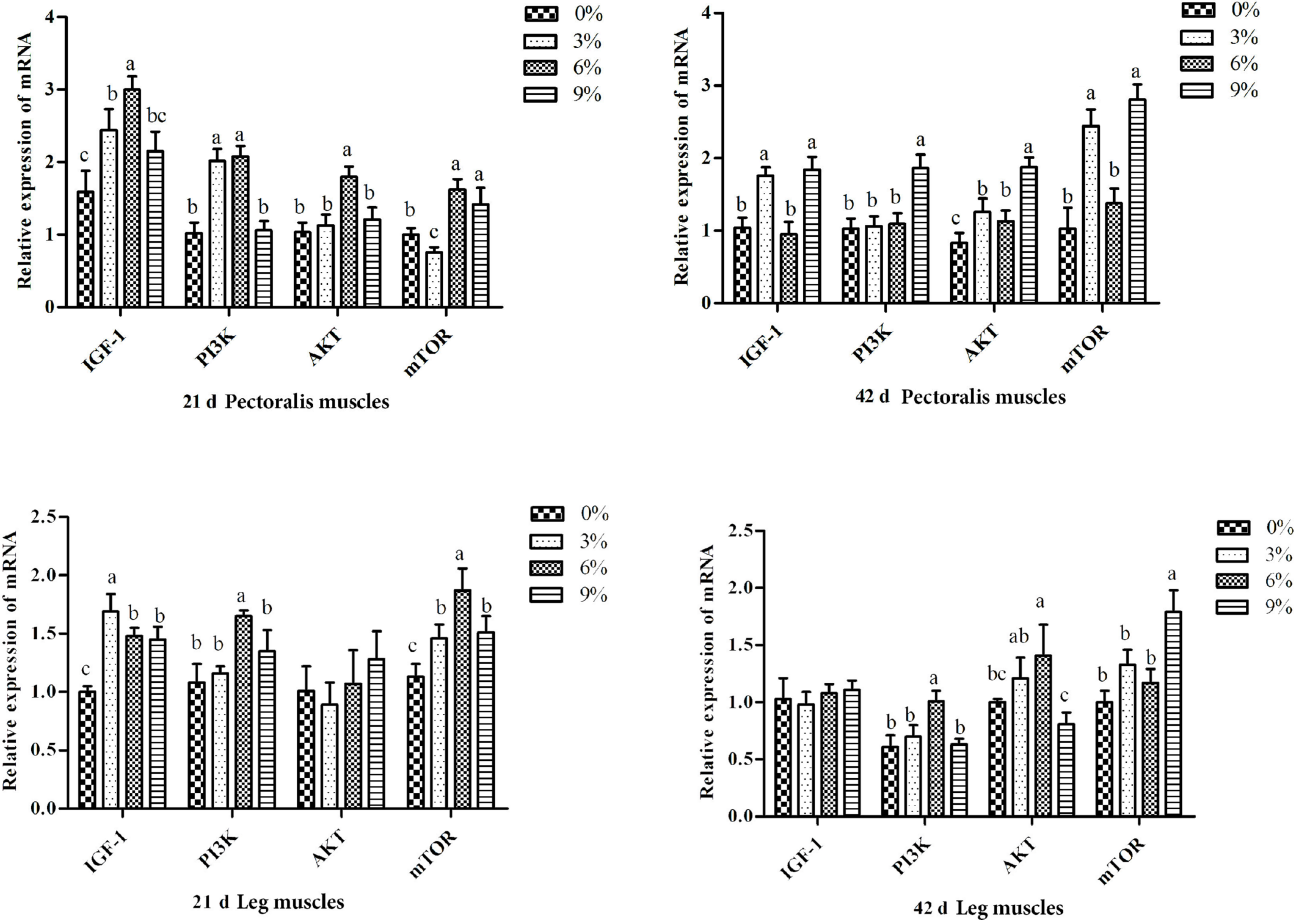
Relative expression of genes in the IGF-1/mTOR pathway of the leg muscles and pectoralis muscles of white-feather broilers on days 21 and 42 (*n* = 6 per group). Different lowercase letters marked above the bars of the gene expression level column chart indicate significant differences (*p* < 0.05), while the same or no letters indicate no significant difference (*p* > 0.05).

As illustrated in Figure 1, during days 1–21, the 3 and 6% fermented cottonseed meal groups exhibited significantly higher mRNA expression of IGF-1 and PI3K in pectoral muscle compared with the control group (*p* < 0.05). Additionally, AKT and mTOR expression in pectoral muscle was significantly upregulated in the 6% group, and mTOR expression was also significantly elevated in the 9% group relative to the control (*p* < 0.05). In leg muscle, IGF-1 and mTOR expression was significantly increased across all treatment groups versus the control (*p* < 0.05), while PI3K expression in the 6% group was significantly greater than that in all other groups (*p* < 0.05). During days 22–42, AKT expression in pectoral muscle was significantly higher in all experimental groups compared with the control (*p* < 0.05). Furthermore, IGF-1 and mTOR expression in pectoral muscle was significantly upregulated in the 3 and 9% groups, and PI3K expression in the 9% group was significantly higher than in all other groups (*p* < 0.05). In leg muscle, PI3K and AKT expression in the 6% group, as well as mTOR expression in the 9% group, were each significantly greater than in the control group (*p* < 0.05); notably, mTOR expression in the 9% group was also significantly higher than in all other treatment groups (*p* < 0.05). No statistically significant differences were observed among the remaining comparisons.

### 3.5 Effects of fermented cottonseed meal on mRNA expression of AMPK pathway genes in protein catabolism of white-feather broilers

**Figure 2:**
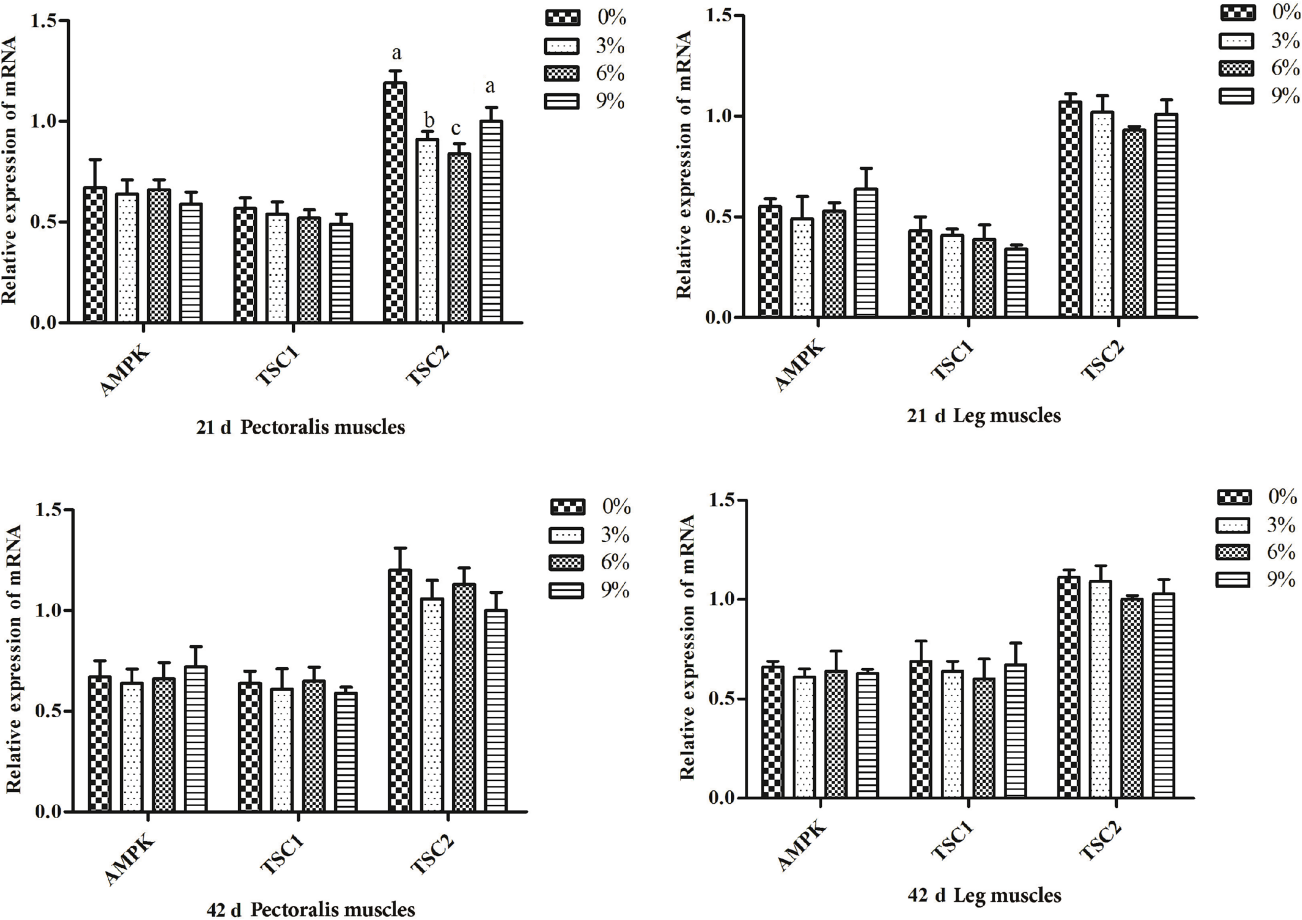
Relative expression of genes in AMPK pathway in the leg muscles and pectoralis muscles of white-feather broilers on days 21 and 42 (*n* = 6 per group). Different lowercase letters marked above the bars of the gene expression level column chart indicate significant differences (*p* < 0.05), while the same or no letters indicate no significant difference (*p* > 0.05).

As shown in Figure 2, the expression levels of AMPK in the pectoral and leg muscles of all experimental groups showed no significant changes compared to that of the control group. Similarly, the addition of fermented cottonseed meal did not significantly affect the expression of TSC1 in the pectoral and leg muscles. During days 1–21, the expression level of TSC2 in the pectoral muscles of the 3 and 6% groups were significantly lower than that in the control group (*p* < 0.05), whereas no significant differences were observed among the other groups.

### 3.6 Effects of fermented cottonseed meal on mRNA expression on UPP pathway gene in protein catabolism of white-feather broilers

**Figure 3:**
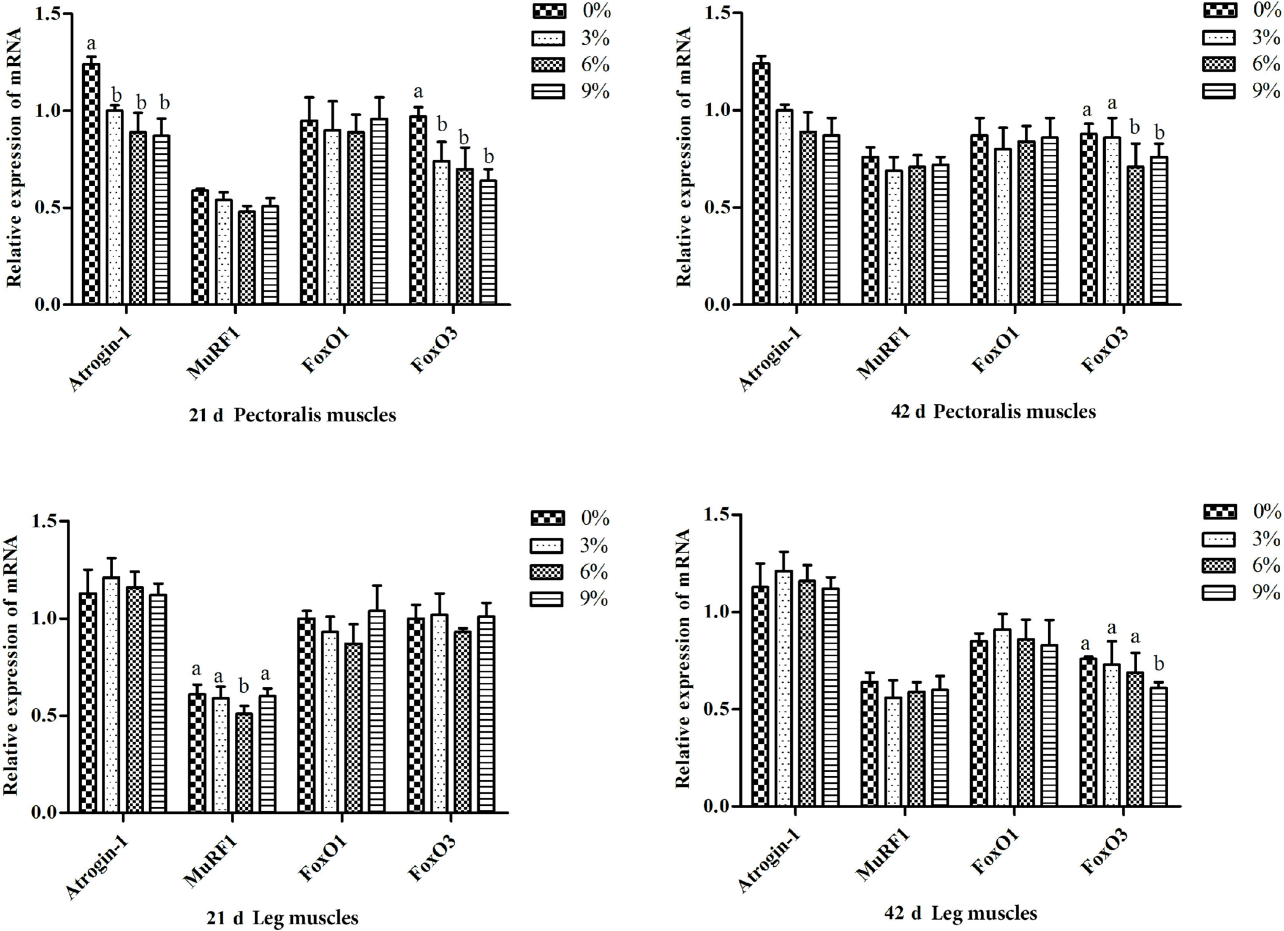
Relative expression of genes in the UPP pathway in the leg muscles and pectoralis muscles of white-feather broilers on days 21 and 42 (*n* = 6 per group). Different lowercase letters marked above the bars of the gene expression level column chart indicate significant differences (*p* < 0.05), while the same or no letters indicate no significant difference (*p* > 0.05).

As illustrated in Figure 3, during days 1–21, mRNA expression of FoxO3 and Atrogin-1 in pectoral muscle was significantly downregulated in all experimental groups relative to the control group (*p* < 0.05). Concurrently, MuRF1 expression in leg muscle was significantly lower in the 6% group compared with both the control group and all other treatment groups (*p* < 0.05). During days 22–42, FoxO3 expression in pectoral muscle remained significantly suppressed in the 6 and 9% groups versus the control (*p* < 0.05), and FoxO3 expression in leg muscle was also significantly reduced in the 9% group relative to the control (*p* < 0.05). No statistically significant differences were observed in any other intergroup comparisons.

### 3.7 Pearson correlation analysis of mRNA expression levels of protein metabolism–associated genes in pectoralis major and iliotibialis muscles with pectoralis major to iliotibialis muscle ratio

**Figure 4:**
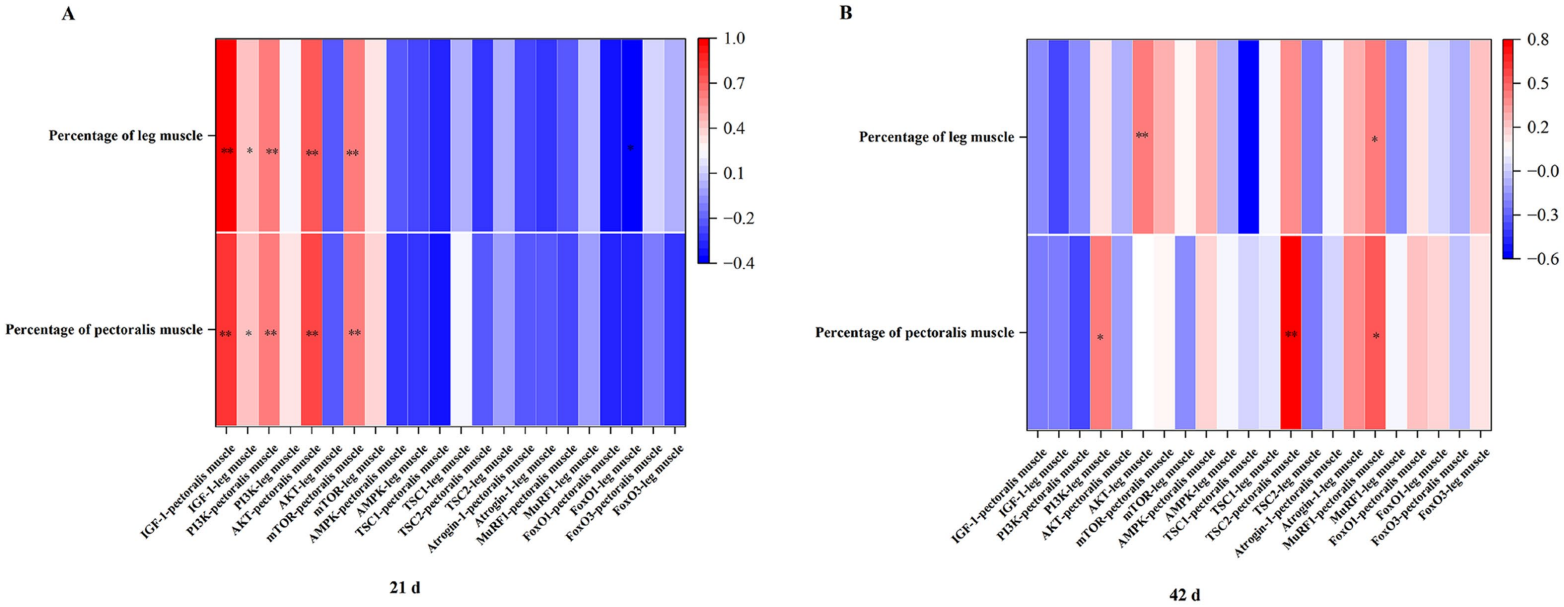
A Pearson correlation analysis chart of mRNA expression levels of genes related to protein metabolism in pectoral muscles and iliotibial muscles and the ratio of pectoral muscles to leg muscles (*n* = 6 per group). Panel **(A)** is a correlation analysis chart of 21 gene expression levels with pectoral muscle rate and leg muscle rate. Panel **(B)** is a correlation analysis chart of 42d gene expression levels with pectoral muscle rate and leg muscle rate. “*” indicates a significant difference between groups (*p* < 0.05), and “**” indicates an extremely significant difference between groups (*p* < 0.01).

As shown in Figure 4, during days 1–21, the mRNA expression levels of IGF-1, PI3K, AKT, and mTOR in the pectoral muscle were all highly significantly correlated with the pectoral muscle rate and leg muscle rate (*p* < 0.01), and the mRNA expression level of IGF-1 in the leg muscle was also highly significantly correlated with the pectoral muscle rate and leg muscle rate (*p* < 0.01), while the mRNA expression level of FoxO1 in the leg muscle was significantly correlated with the leg muscle rate (*p* < 0.05). During days 22–42, the expression level of PI3K in the leg muscle was significantly correlated with the pectoral muscle rate (*p* < 0.05), the expression level of AKT in the leg muscle was highly significantly correlated with the leg muscle rate (*p* < 0.01), the expression level of TSC2 in the pectoral muscle was highly significantly correlated with the pectoral muscle rate (*p* < 0.01), and the expression level of MuRF-1 in the pectoral muscle was significantly correlated with both the pectoral muscle rate and the leg muscle rate (*p* < 0.05). There was no significant correlation among other indicators.

## 4 Discussion

### 4.1 Effects of fermented cottonseed meal on growth and slaughter performance of white-feather broilers

Cottonseed meal, a high-quality protein feed with a crude protein content exceeding 40%, contains free gossypol, which that limits its use in animal feed. However, microbial fermentation effectively addresses this limitation (3, 4). After fermentation, cottonseed meal has enhanced levels of small peptides, amino acids, and organic acids. Feeding broilers with this fermented meal improves production performance, with better results during the early-growth phase than during the late-growth phase (13). Our study revealed that the addition of fermented cottonseed meal during the early-growth phase of white-feather broilers increased the average daily weight gain while reducing the feed-to-weight ratio, with the 6% inclusion level producing the best outcomes. This may be because fermentation reduces toxic components such as free gossypol; however, at a higher inclusion level (9%), residual gossypol or other fermentation-derived by-products (e.g., certain microbial metabolites) may accumulate and induce mild stress in the birds, thereby partially offsetting the beneficial effects. Previous studies have also indicated that probiotics in broiler feed enhance digestive enzyme activity (14, 15). The fermented cottonseed meal used in this experiment contained 2.13 × 10^6^ CFU/g of live *Candida tropicalis*. These probiotics colonize the digestive tract, secrete enzymes to improve feed utilization, and consequently enhance production performance (16). Additionally, the high organic acid content of fermented cottonseed meal help to regulate intestinal pH, inhibits the growth of harmful microorganisms, and improves digestive health and efficiency (17, 18). Furthermore, we observed inconsistencies in the changes of growth performance indicators (F/G and ADFI) between the early (1–21 days) and late (22–42 days) stages. We believe that these stage-specific differences may originate from variations in the digestive system development and metabolic demands of broilers at different growth phases. During days 1–21, the gut and microbiota are still being established, making broilers more responsive to dietary interventions; probiotics/enzymes and organic acids in fermented cottonseed meal may enhance digestion and nutrient absorption, thereby significantly reducing F/G. In contrast, during days 22–42, digestive function becomes more stable and metabolism shifts toward intensive muscle deposition, so the marginal benefits diminish; moreover, higher inclusion may increase dietary fiber and dilute nutrient density, limiting further improvements and leading to more variable ADFI trends.

The pectoral muscle ratio and leg muscle ratio, as core indicators reflecting the body’s protein metabolism level and muscle deposition efficiency, are directly associated with the meat production performance and muscle quality of broilers. Studies have confirmed that the addition of microbially fermented cottonseed meal to the basal diet of broilers can degrade anti-nutritional factors such as gossypol in cottonseed meal and improve the digestibility and absorption rate of crude protein and amino acids. This not only significantly enhances the overall growth performance of broilers and increases the average daily gain, but also directionally optimizes the muscle tissue deposition pattern, thereby effectively increasing the pectoral muscle ratio and leg muscle ratio (19, 20). Our experimental results corroborate these findings; the 6% supplementation group showed significantly higher pectoral and leg muscle proportions than the control group during the early-growth phase. Additionally, both the 3 and 6% supplementation groups exhibited markedly higher carcass yields and total clean weights than the control group. These results indicate that fermented cottonseed meal enhances protein metabolism and accelerates muscle growth in broiler chickens. Nie et al. (5) further confirmed that fermented cottonseed meal improves endogenous metabolism by modulating gene expression in metabolic pathways, thereby influencing overall physiological processes.

### 4.2 Effects of fermented cottonseed meal on mRNA expression of genes related to protein metabolic synthesis pathways in white broiler chickens

The synthesis and catabolism of proteins in animals are regulated by multiple factors, including environmental conditions, hormonal status, and nutritional status. Studies have shown that the use of fermented cottonseed meal in broiler feed can enhance the pectoral and leg muscle ratios (7) while improving carcass protein deposition capacity (8). During this process, the mTOR signaling pathway regulates protein synthesis within the muscle cells (21). IGF-1, a key member of the IGF family located on chromosome 12, is a critical growth-promoting factor (22, 23). It plays a vital role in promoting cell proliferation and differentiation and skeletal muscle protein synthesis (24). Mechanistically, IGF-1 activates protein synthesis through the phosphorylation of the mTOR signaling pathway (25) while also enhancing ornithine decarboxylase activity to promote protein, DNA, and RNA synthesis, ultimately inducing cell proliferation (26). Additional research has indicated that IGF-1 can induce muscle cells to enter new cell cycles, thereby accelerating their proliferation and muscle fiber formation (24). The addition of IGF-1 to myoblast and myotubule culture systems significantly increases protein synthesis rates while inhibiting degradation (27). The experimental results demonstrated that supplementing fermented cottonseed meal into the diet of white-feather broilers significantly enhanced IGF-1 expression in muscle tissue, with more pronounced effects observed in the early-growth phase than in the late-growth phase. Notably, the 6% supplementation group showed superior performance compared to the 3 and 9% groups. Therefore, moderate supplementation of fermented cottonseed meal in broiler feed may promote protein synthesis in muscle tissues by upregulating IGF-1 expression.

After binding to its receptor, IGF-1 activates the PI3K signaling pathway, thereby promoting cell proliferation and inhibiting apoptosis (28, 29). Our experimental results demonstrate that during the early-growth phase of broilers, the expression levels of PI3K and IGF-1 mRNAs in the pectoral muscles of the 6% supplementation group showed synchronous upward trends. As a key regulatory node in protein synthesis and catabolism, mTOR influences protein metabolism by regulating downstream molecular expression (30). Specifically, when IGF-1 binds to its receptor, it activates PI3K. Activated PI3K catalyzes the formation of PI-3,4,5-P3, which subsequently activates phosphatidylinositol-dependent kinase-1 (PDK-1), leading to AKT phosphorylation. Phosphorylated AKT further activates the upstream regulators of mTOR, thereby initiating the mTOR pathway and ultimately regulating protein metabolism (31, 32). This study indicated that the addition of fermented cottonseed meal to broiler diets significantly enhanced the mRNA expression levels of AKT and mTOR in muscle tissues, with the 6% supplementation group showing the most significant effects compared to the 3 and 9% supplementation groups. Moreover, the magnitude of upregulation during the early-growth phase was greater than that during the late-growth phase. These findings suggest that fermented cottonseed meal may regulate protein anabolism by influencing the mRNA expression of genes related to the IGF-1/mTOR pathway. The abundance of microbial metabolites in fermented cottonseed meal likely explain its primary role in regulating protein synthesis in the pectoral and leg muscles through the IGF-1/mTOR pathway. Although the energy and crude protein levels of diets in all groups were consistent in this study, the arginine content in fermented cottonseed meal was higher than that in soybean meal (11). Previous studies have demonstrated that arginine can enhance protein synthesis and suppress protein degradation in small intestinal epithelial cells by upregulating the mRNA expression of genes associated with the mTOR pathway (33). Additionally, in a previous study, we revealed increased levels of metabolites such as peptides and succinic acid in fermented cottonseed meal substrates (34), all of which have been reported to enhance protein synthesis (35, 36). Other studies have indicated that fermented cottonseed meal can also elevate growth hormone and thyroxine (T4) levels in broiler pectoral and leg muscles, as well as increase the activity of lactate dehydrogenase and alkaline phosphatase (7). These findings suggest that hormonal and enzymatic changes in muscle tissue may indirectly regulate protein synthesis by influencing the expression of genes, such as mTOR. However, given the complex composition of fermented cottonseed meal, the detailed mechanisms of action require further investigation. This study demonstrates that the growth and development of muscle tissues in broilers are orchestrated by an integrated systemic regulatory network. During the early growth phase, the expression levels of key genes in the IGF-1/PI3K/AKT/mTOR pathway in the pectoralis major not only exhibit a significant positive correlation with the pectoralis major muscle ratio but also show a strong correlation with the leg muscle ratio. This suggests that the activation of this pathway in the pectoralis major can serve as a sensitive molecular marker reflecting the early systemic anabolic state of the organism. In the late growth phase (days 22–42), the regulatory pattern shifts: the expression of PI3K and AKT in the leg muscles correlates significantly with the pectoralis major muscle ratio and leg muscle ratio, respectively, indicating prominent cross-regulatory interactions between different muscle tissues. This finding aligns with the previously proposed mechanism that key metabolic genes in muscle can modulate muscle development via systemic signal transduction (37). Collectively, this study uncovers the integrative and coordinated nature of muscle growth in broilers at the gene expression level, providing a theoretical foundation for the synchronous enhancement of meat production performance through nutritional or genetic strategies.

The IGF-axis transcriptional responses to FCM supplementation appeared to be both tissue- and age-dependent. At 21 days, IGF pathway–related genes were generally upregulated in both pectoral and leg muscles, suggesting that dietary modulation may more readily engage anabolic signaling during the early growth phase when the digestive system and nutrient utilization capacity are still maturing. By 42 days, this upregulation was not consistently maintained, particularly in pectoral muscle where inter-group variability increased. Such heterogeneity may reflect phase-specific growth dynamics in broilers (rapid early accretion vs. a more plateaued finishing phase), as well as intrinsic differences between muscle types. Pectoral muscle is predominantly fast-twitch glycolytic, whereas leg muscle contains a higher proportion of oxidative fibers and experiences continuous mechanical loading, which may confer distinct regulatory sensitivity and baseline signaling. Collectively, these features could explain why leg muscle maintained relatively stable expression patterns at moderate inclusion levels, while pectoral muscle showed greater variability at 42 days.

### 4.3 Effects of fermented cottonseed meal on the mRNA expression of genes related to the protein metabolism pathway of white-feather broilers

The activation of mTOR is regulated by multiple signaling pathways, among which AMPK exerts negative regulatory effects (38). When an organism faces stress or an insufficient energy supply, AMPK is activated to alleviate the physiological damage caused by stress. Studies have shown that under glucose-deficient stress conditions, intracellular ATP levels decrease, whereas AMP levels increase, inducing allosteric modulation and phosphorylation of AMPK (39). Activated AMPK subsequently promotes the uptake and metabolism of fatty acids and glucose while inhibiting protein synthesis processes (40). The experimental results indicated that the addition of fermented cottonseed meal did not significantly affect AMPK mRNA expression in broiler pectoral and leg muscles, suggesting that changes in AMPK at the transcriptional level are not the primary mechanisms influencing protein metabolism in broilers. However, AMPK may indirectly suppress mTOR activity and downstream protein synthesis-related molecular expression by phosphorylating TSC1/2 proteins, thereby reducing the protein synthesis rates (41). This study also showed that TSC2 expression is downregulated in broiler pectoral muscles during the growth phase, indicating that AMPK may participate in the regulation of protein catabolism through downstream gene modulation.

In most animal organisms, protein turnover and renewal are primarily regulated by the ubiquitin-proteasome pathway (UPP), which accounts for 40–50% of protein degradation in eukaryotes (42). The UPP ubiquitinates target proteins through a three-step enzymatic reaction: first, the ubiquitin ligase (E1) activates ubiquitin molecules; then, ubiquitin is transferred to the ubiquitin ligase (E2), and finally, ubiquitin is covalently attached to specific target proteins by the ubiquitin ligase (E3). Ubiquitinated target proteins are recognized by the 26S proteasome and degraded into amino acids and small peptides (43). During this process, the ubiquitin-target protein interaction is mediated by tissue-specific E3 ligases, and their binding efficiency constitutes the rate-limiting step of the UPP. Studies have shown that Atrogin-1 and MuRF1 are two specifically expressed E3 ligases in skeletal muscle (44). In the present study, the addition of 6% fermented cottonseed meal significantly reduced the mRNA expression levels of Atrogin-1 and MuRF1 in broiler muscle, suggesting that fermented cottonseed meal may slow protein degradation by inhibiting E3 ligase expression. In contrast, the forkhead transcription factor (FOXO) family, which are evolutionarily conserved proteins, participate in processes such as promoting protein degradation and inhibiting tumor growth (44). Notably, FOXO1 and FOXO3 can enhance muscle atrophy by upregulating the expression of Atrogin-1 and MuRF1 (45). The findings of this study revealed that the regulatory effects of fermented cottonseed meal (FCM) on FOXO3, Atrogin-1, and MuRF1 in the pectoralis major and leg muscles of broilers exhibit tissue-, time-, and dose-dependency. Specifically, in the pectoralis major muscle of 21-day-old broilers, dietary supplementation with 3, 6, and 9% FCM significantly downregulated the mRNA expression of FOXO3 and Atrogin-1. At 42 days of age, only the 6 and 9% FCM groups induced a significant downregulation of FOXO3 in the pectoralis major muscle, while a significant reduction in FOXO3 expression in the leg muscle was solely observed in the 9% FCM group at 42 days of age. Notably, MuRF1 mRNA levels were significantly decreased only in the 6% FCM group of 21-day-old broilers’ leg muscle. Moreover, the results of the correlation analysis indicated that the down-regulation of MuRF1 expression was significantly correlated with the growth of the pectoral and leg muscles. Previous research has established that the FOXO family, particularly FOXO1 and FOXO3, can bind to the promoters of Atrogin-1 and MuRF1, thereby accelerating myofibrillar protein degradation via the ubiquitin-proteasome pathway (46). The synchronous downregulation of FOXO3 and Atrogin-1 in the pectoralis major muscle of 21-day-old broilers in the present study further validates their transcriptional regulatory relationship. Collectively, these results indicate that appropriate dietary supplementation with FCM can suppress the transcriptional activity of FOXO3 in skeletal muscle, selectively downregulate the expression of Atrogin-1 and MuRF1, and consequently attenuate muscle protein degradation while promoting net protein accretion. The specificity of this regulatory effect may be associated with the metabolic characteristics of broiler skeletal muscle and the utilization efficiency of bioactive components in FCM. These mechanistic insights provide a theoretical basis for optimizing FCM supplementation strategies in broiler production based on different growth stages.

FCM supplementation was associated with a marked reduction in the expression of atrophy-related factors at 21 days, whereas fewer significant differences were detected at 42 days. Importantly, baseline expression of catabolic/atrophy-related genes is typically low during early growth and tends to increase with age; therefore, suppression observed at the finishing stage may carry greater biological relevance. Accordingly, the early-stage downregulation observed here should be interpreted as an indication of early modulation of catabolic signaling rather than definitive evidence of functional “atrophy prevention.” The absence of broad significance at 42 days may reflect a reduced effect size and/or increased biological variability during the finishing phase.

In addition, because the present study primarily reports mRNA-level changes, the mechanistic interpretation should remain cautious in the absence of protein-level validation. Future work incorporating protein measurements and pathway activation markers would help further substantiate the proposed regulatory relationships.

In summary, fermented cottonseed meal can coordinately regulate the expression of genes associated with protein synthesis and degradation in the skeletal muscle tissue of white-feathered broilers. With respect to protein synthesis, particularly during the early growth phase and at a 6% dietary inclusion level, it significantly upregulates the mRNA expression of key components in the IGF-1/PI3K/AKT/mTOR signaling pathway. In terms of protein degradation, it concurrently suppresses the transcription of upstream regulatory factors in the ubiquitin–proteasome pathway FoxO1 and FoxO3 as well as their downstream effector molecules, Atrogin-1 and MuRF1. These coordinated yet opposing regulatory effects promote enhanced net protein accretion, which aligns with the observed improvements in growth performance and slaughter traits. Thus, it can be inferred that fermented cottonseed meal may modulate the gene regulatory network of muscle protein metabolism through multiple targets, potentially mediated by its bioactive fermentation products. Nevertheless, the precise cascade regulatory mechanism warrants further investigation at the levels of post-translational modifications and protein–protein interactions.

## 5 Conclusion

In conclusion, fermented cottonseed meal enhances protein synthesis in the pectoral and leg muscles of broiler chickens by upregulating genes in the IGF-1/mTOR signaling pathway, while simultaneously inhibiting protein degradation via the UPP pathway. The 6% supplementation level demonstrated the most significant effects, with greater regulatory efficacy observed during the early-growth phase (1–21 days) than during the later-growth phase (22–42 days).

## Data Availability

The original contributions presented in the study are included in the article/supplementary material, further inquiries can be directed to the corresponding authors.
